# S14-1: EUPAP Feasibility Study. Practice transfer of a HEPA prescription model to other nine EU countries. From theory to practice

**DOI:** 10.1093/eurpub/ckae114.260

**Published:** 2024-09-26

**Authors:** Sebastià Mas-Alòs, Bruno Avelar Rosa, Dempsey Demeyer, Carmen Domnariu, Estela Farías-Torbidoni, Cristina Godinho, Lena Hansson, Luc Lipkens, Sergi Matas, Laura Merlo, Antoni Planas-Anzano, Catarina Santos Silva, Ciprian Ursu, Ioana Vestemean, Marita Friberg

**Affiliations:** National Institute of Physical Education of Catalonia (INEFC); General-Directorate of Health, Ministry of Health, Portugal; Flemish Institute of Healthy Living, Flanders, Belgium; National Institute of Public Health (NIPH), Romania; National Institute of Physical Education of Catalonia (INEFC); General-Directorate of Health, Ministry of Health, Portugal; Public Health Agency of Sweden, Solna, Sweden; Flemish Institute of Healthy Living, Flanders, Belgium; National Institute of Physical Education of Catalonia (INEFC); Azienda Unita Locale Socio Sanitaria n2 Marca Trevigiana, Treviso, Italy; National Institute of Physical Education of Catalonia (INEFC); General-Directorate of Health, Ministry of Health, Portugal; National Institute of Public Health (NIPH), Romania; National Institute of Public Health (NIPH), Romania; Public Health Agency of Sweden, Solna, Sweden

## Abstract

**Purpose:**

The Swedish Physical Activity on Prescription (PAP-S) method was to be transferred to other European regions through the EUPAP Project, innovative for implementing a reliable method to other nine countries. Implementation practices are strongly context-dependent; therefore, the practice transfer requires a flexible approach.

PAP-S results over 20 years have reported success to a variety of populations. It addresses physically inactive individuals and/or with chronic conditions to improve their health by increasing their physical activity (PA) levels. The PAP-S five core components are: individualised patient-centred counselling, evidence-based PA recommendation, written prescription, community support, and follow-up.

The goal was to know the context regarding PA prescription and ease the transfer from the Swedish context, specifically to: a) setting relevant PAP-S indicators for planning and monitoring, b) creating guidelines for data collection, c) providing an overview of the situation in each region, and d) assessing for readiness to implement the model.

**Project description:**

Modified Hybrid e-Delphi process by representative from 10 countries. First, commitment from organisations to assume costs for the implementation. Second, description of variables and procedures for data collection. Data comprised two dimensions (ie, macro level (early diagnosis) and micro level (preparedness for implementation)). Third, data collection. Fourth, data analysis and discussion.

Thirty-five experts participated in the EUPAP Feasibility Study. Guidelines for data collection includes indicators on macro level: a) EUPAP-relevant policy documents, b) profiles of prescribers and allied professionals, c) programmes, materials and training on HEPA, d) regulations, e) budget; and micro level: a) stakeholders, b) settings, c) agents, d) end-users.

Country-specific results were collected, compared and discussed in relation to the core components of PAP-S (findings are accessible on www.eupap.org). Project partners disseminated the results among region-specific stakeholders.

**Conclusions:**

Data showed regions with solid background to launch the EUPAP implementation (Flanders, Catalonia, Portugal) and others with less existing materials or weak network (Malta, Romania, Germany). Policy-makers, local health services and communities may better set short- mid- and long-term goals for the PAP-S transfer. Further design on practices and policies may use the Feasibility Study Guidelines for setting specific procedures.

**Funding:**

Co-funded by the European Union’s Health Programme (2014-2020).

